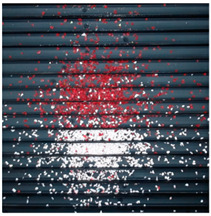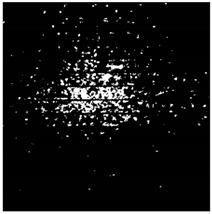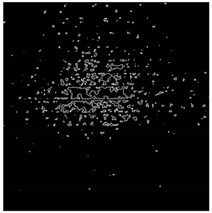# Assessment of the Electrostatic Separation Effectiveness of Plastic Waste Using a Vision System

**DOI:** 10.3390/s20247201

**Published:** 2020-12-16

**Authors:** Dominik Rybarczyk, Cezary Jędryczka, Roman Regulski, Dariusz Sędziak, Krzysztof Netter, Dorota Czarnecka-Komorowska, Mateusz Barczewski, Mariusz Barański

**Affiliations:** 1 Institute of Mechanical Technology, Poznan University of Technology, 60-965 Poznań, Poland; roman.regulski@put.poznan.pl (R.R.); dariusz.sędziak@put.poznan.pl (D.S.); krzysztof.netter@put.poznan.pl (K.N.); 2 Institute of Electrical Engineering and Electronics, Poznan University of Technology, 60-965 Poznań, Poland; cezary.jedryczka@put.poznan.pl (C.J.); mariusz.baranski@put.poznan.pl (M.B.); 3 Institute of Materials Technology, Poznan University of Technology, 60-965 Poznań, Poland; dorota.czarnecka-komorowska@put.poznan.pl (D.C.-K.); mateusz.barczewski@put.poznan.pl (M.B.)

**Keywords:** vision system, plastics recycling, PS, PMMA, electrostatic separation effectiveness

## Abstract

The work presented here describes the first results of an effective method of assessing the quality of electrostatic separation of mixtures of polymer materials. The motivation for the research was to find an effective method of mechanical separation of plastic materials and a quick assessment of the effectiveness of the method itself. The proposed method is based on the application of a dedicated vision system developed for needs of research on electrostatic separation. The effectiveness of the elaborated system has been demonstrated by evaluating the quality of the separation of mixtures of poly (methyl methacrylate) (PMMA) and polystyrene (PS). The obtained results show that the developed vision system can be successfully employed in the research on plastic separation, providing a fast and accurate method of assessing the purity and effectiveness of the separation process.

## 1. Introduction

One of the key problems of mechanical recycling of plastic wastes is the development of effective methods for their separation [1]. Many research teams in Poland and all over the world, undertake intensive efforts to provide low-cost technologies with a high throughput and quality of selective sorting of various plastics in mixtures that are a result of waste management and segregation in a circular economy [2,3]. A circular economy has the task of treating plastic waste as valuable raw materials circulating in the industrial economy and incorporating them into the life cycle of subsequent products, i.e., the flow of non-renewable resources in closed cycles [3].

**Figure 1:**
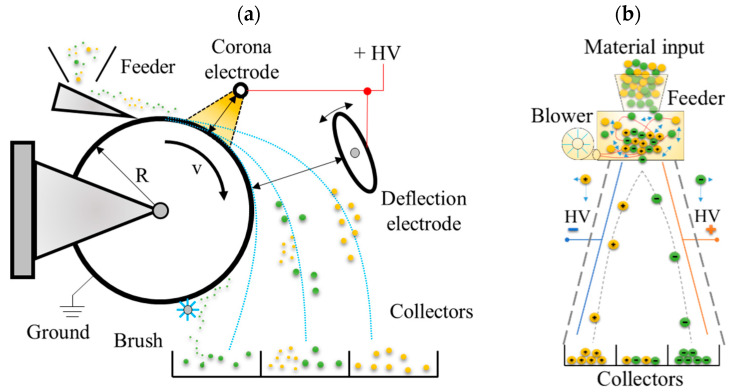
Illustration of electrostatic separation process in the example of a drum type separator (**a**) and free-fall type (**b**).

Sink-flotation is one of the most commonly applied methods for separating plastics in mechanical recycling [4,5]. Despite the simplicity and low cost of processing, this method is not effective for plastics with similar density. To separate mixtures of so-called “difficult materials” having a similar density such as acrylonitrile-butadiene-styrene (ABS), PS and PMMA, more complex methods must be developed. Electrostatic separation (ES) offers an opportunity to separate polymeric materials of the same or very similar density [6,7]. The principle of electrostatic separation is based on differences in electrostatic forces acting on particles of the mixture exposed to the electric field [6,8]. The differences in the electric forces can be caused by the effect of (a) the difference of charge decay of the sorted materials [9] and (b) by the different polarization of the charge in the particles of particular materials [8,10]. Utilization of (a) or (b) effects require different constructions of the separator. When the difference in charge decay takes effect, the particles of the plastic mixture are charged with the same polarity, usually using the corona discharge electrode. The most common structures of separators utilizing this effect are drum and belt type separators [11,12,13,14] shown in Figure 1a. The positive and negative charging of the particles in the plastic mixture is achieved by the tribocharging process performed in special devices called tribochargers. Next, plate-type separators (Figure 1b) are used to segregate the charged materials. The availability of effective numerical methods such as the finite element method, allowing one to predict the particle trajectories in studied systems, [14,15,16,17,18] has led to a high number of designs being intensively investigated in past decades.

Nevertheless, despite the availability of accurate numerical models and simulation tools, corona charging as well as tribocharging of plastic materials are very complex physical processes depending on many factors that cannot be easily predicted at the design stage. The effectiveness of electrostatic separation depends, among others, on the size and shape of particles in the mixture (which has a stochastic character), environmental conditions like humidity, pressure and temperature, the moisture content of the mixture as well as its voltage level, the configuration of the electrode system, and the position of the feeding unit. Summarizing the above, it can be concluded that despite the availability of numerical models and simulation tools, the important part of the research on electrostatic separation are empirical tests performed on dedicated laboratory test benches. In this respect, looking at a high number of parameters affecting the separation process, effective methods to assess the purity of the separation are needed for the research on electrostatic separation. The desired method should provide fast and reliable results to enable the optimization of parameters of the separation process.

## 2. Methods of Assessment of Purity and Effectiveness of Electrostatic Separation Process

In order to increase the efficiency of sorting techniques of mixed plastics, various sensors that recognize plastics based on colour, visual image spectroscopy (VIS), hyperspectral imaging (HIS) or X-rays, and electromagnetic electron (EM) sensors are used [19,20]. For example, mixed plastics can be recognized based on their colours. The capabilities of these sensors extend beyond the visible light spectrum and include detection in infrared, ultraviolet, and other frequency ranges [1,3,19].

The following optic sensor-based separation devices were used: near infrared (NIR) [3,21], a colour line camera, X-ray fluorescence [19,22,23]. The optic sensor equipment separates materials such as individual plastic polymers, e.g., polyethylene PE, poly(vinyl chloride) PVC, poly(ethylene terephthalate) PET, ABS and colours. However, black plastic particles cannot normally be separated as they don’t give a reflection (poor reflectance of infrared light) [23]. Next, UV sensors (photodiodes) recognize the plastics via the individual wavelength known for each polymer and air jets subsequently sort them into different compartments. The NIR separator is typically applied for separation of mixed thermoplastics by polymer type (except black-coloured grades) [19,22,23]. The efficiency of NIR separators for separation from the input stream and purity of the output stream depends on the variety of the material. The plastic separation efficiency of NIR-based detection systems is about 80–90%. Typically, each run of NIR sorting can sort by two parameters (output of two fractions). Sorting by more than three parameters thus requires more runs or more NIR scanners, which causes decreasing effectiveness of the process [23]. The colour line camera system is usually used to separate different colours, but not polymeric materials (e.g., bottles). The dissemination of this method would significantly improve the separation efficiency of mixed materials, but the cost of this technology is still high and similar to the NIR technology. Furthermore, X-ray fluorescence separation (XRF) is a type of automatic, sensor-based sorting, using X-ray photons to impact a targeted material and detect movements of electrons followed by emission of X-rays. X-ray fluorescence separation is not, at present, widely used when sorting mixed waste, as this technology is not able to distinguish between polymers [23].

Industrial solution devices such as the near infrared ‘Polysort’ sorter and near infrared ‘Unisort’ sorter are known for separating by polymer type [24]. However, the first of them was only able to identify and separate black plastic materials consisting of larger particles (greater than 6 mm). In the second case, although the proposed system can identify material using NIR, it is not able to distinguish between ABS and PS, or differentiate them from relatively light particles. Additionally, systems of this type provide relatively limited data (only eight results on) polymer spectra at a time, which is definitely too little to sort complex mixtures of cable waste [23].

**Figure 2:**
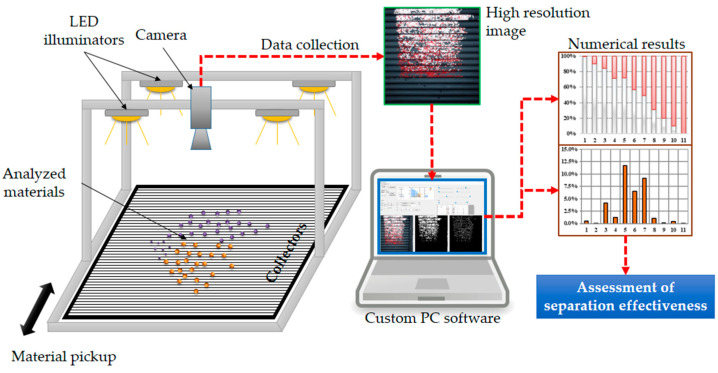
Concept of the vision system for the assessment of ES effectiveness.

An alternative method employing a dedicated vision system in the research on the separation of polymers is proposed. The concept is based on methods that use samples of materials of a different colour. The idea is schematically illustrated in Figure 2. The system consists of a segmented collector of the separated material, a camera, a lighting system and a PC with a developed computer code.

The task for the proposed vision system is to assess the purity of the material in each segment (row of the collector) based on the image taken by a camera. The developed computer code must provide numeric data about the amount of each material in the row. The data are then processed to calculate the separation efficiency factor.

## 3. Vision System for the Assessment of ES Effectiveness

The advantages of vision systems are utilized in the rapid and reliable assessment of many technological processes. The colour classification for wooden boards was reported in [25]. The article describes the use of HSV (Hue, Saturation, Value) filtration to classify colours of wooden boards. This information is also used to remove background and varnish colours. The feature extraction method is based on a 3D colour histogram. The obtained data was taken into account in further analysis. The authors of [26] estimated the size and mass of fruit and vegetables based on a single top view image. Experimental results showed good results for the chosen products. The mean volume estimation error was about 3%. Based on the RGB images, the biomass quality was also assessed in the article [27].

**Figure 3:**
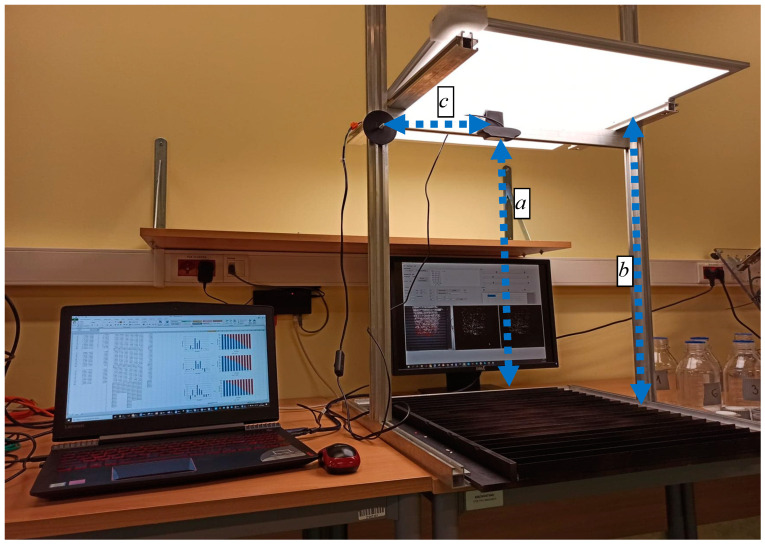
Test rig and components of the developed vision system.

**Figure 4:**
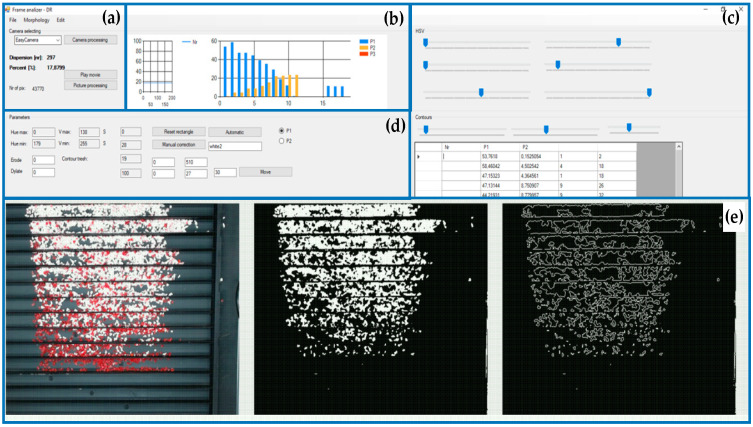
Material pickup consisting of 18 regions of interest (ROIs) filled with sample materials: (**a**) camera selection, (**b**) data visualization (preview), (**c**) HSV colour settings, (**d**) ROI parameter selection, (**e**) camera view (before and after image processing).

The concept of utilizing a vision system for the rapid assessment of electrostatic separation effectiveness is mainly based on using dedicated sample materials that differ in colour. The mixture of these sample materials is used to study the impact of the process parameters on the quality of this separation. Such a fast method of assessing the effectiveness of the separation, provided by the vision system, allows the determination of “optimal parameters” in the process of separating selected materials. The proposed vision system for the rapid assessment of the separation effectiveness is based on a CMOS camera and in-house developed software running on a standard PC. The camera resolution was 1920 × 1080 px. The codec H.264 was used for compression. The camera was installed on a frame made of aluminium profiles, thanks to which it was possible to adjust its field of view. To minimize the impact of ambient light conditions the working space was illuminated with a 20W LED panel. The test stand had the following dimensions: c = 300 mm, a = 600 mm, b = 670 mm—see Figure 3. The separated material was placed in a number of collectors (see Figure 4) made of aluminium C-profiles forming a material pickup. For testing purposes, in order to avoid reflections, the collectors were painted with matte black paint. The test rig and components of the developed vision system have been shown in Figure 3.

The main task of the developed system is to estimate the number of materials in each collector, called, in this context, a region of interest (ROI). The material pickup, shown in Figure 4, consists of 18 collectors. The collectors are filled with the sample materials during the separation process when the material pickup is connected to the electrostatic separator.

The application of the most frequently used RGB colour model to evaluate the amount of material of a given colour is not convenient. The main reason is the disadvantage of the RGB model related to the dependence of the R, G and B components on lighting conditions. This is due to the fact that all of the components of this model depend on lighting conditions. To mitigate this problem, it is beneficial to use the HSV colour space instead of the RGB model. Thanks to the colours in the HSV model, represented as points lying on and inside the cone in accordance with Formulas (1)–(3) [28,29], it is much easier to determine the ranges of HSV parameters for materials of specific colours. Moreover, the recognition of materials by their colours is much less dependent on lighting conditions. Therefore, the proposed approach used the HSV colour model for image analysis. Thanks to the application of the HSV colour model, materials of very similar colours can be distinguished even under different lighting conditions.

(1) $$H=\left\{\begin{array}{cc} 60^{o}\times \frac{\left[R-B\right]}{Max\left(R,G,B\right)-Min\left(R,G,B\right)} & if\;Max\left(RGB\right)=R\\ 60^{o}\times \frac{\left[B-R\right]}{Max\left(R,G,B\right)-Min\left(R,G,B\right)}+120^{o} & if\;Max\left(RGB\right)=G\\ 60^{o}\times \frac{\left[R-G\right]}{Max\left(R,G,B\right)-Min\left(R,G,B\right)}+240^{o} & if\;Max\left(RGB\right)=B \end{array}\right.\;$$

(2) $$S=[\frac{Max\left(R,G,B\right)-Min\left(R,G,B\right)}{Max\left(R,G,B\right)}]\;$$

(3) $$S=[\frac{Max\left(R,G,B\right)-Min\left(R,G,B\right)}{Max\left(R,G,B\right)}]$$

**Figure 5:**
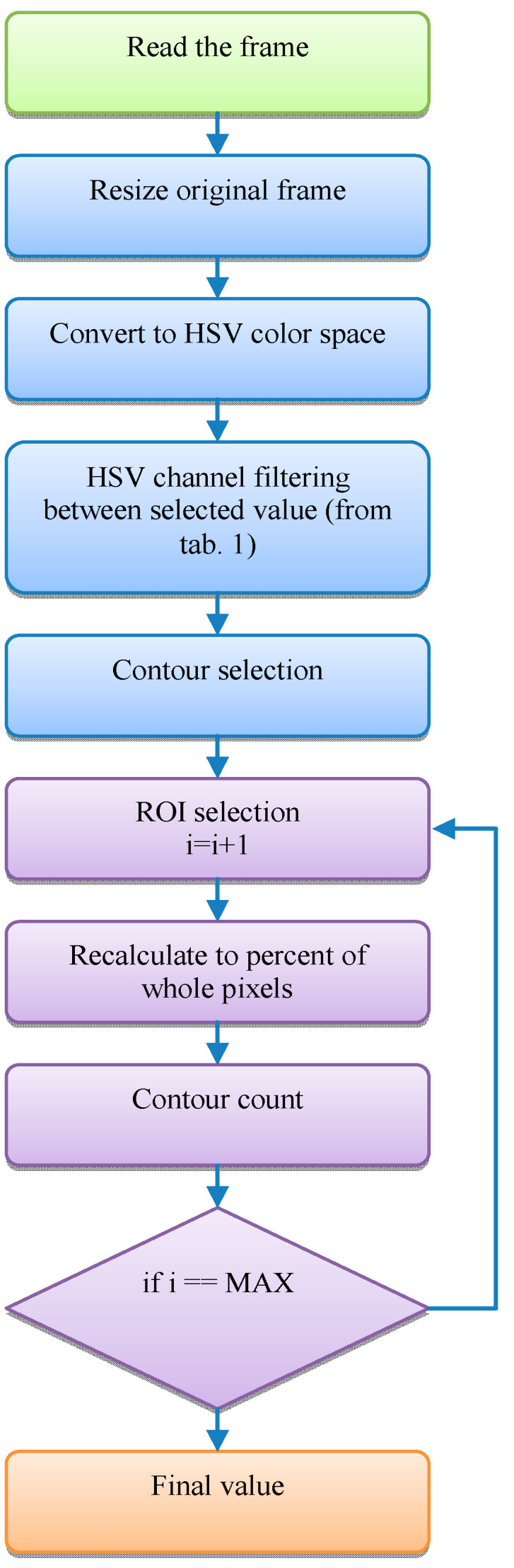
Image processing algorithm.

The in-house code for image processing was written in the C# programming language using OpenCV libraries. During the first stage of the development of the system, an algorithm was calibrated and the individual HSV data for a specific material and lighting conditions were determined. After the image is loaded, the defined segment ROI is cut out for further analysis. The dimensions of a single ROI are 510–27 px. Then, each ROI is filtered using previously calibrated values taken from the HSV colour model. During the next step the numerical representation of the material content is calculated. Two indicators were used for the assessment of the separation effectiveness. The first is defined by the percentage of pixels with a certain colour occupying a given image segment (ROI). The second parameter describes the number of contours found in the filtrated image. This parameter was defined as the degree of dispersion. Thanks to a high degree of dispersion it was possible to notice that a given material was clearly grouped in specific zones. Nevertheless, it should be treated as an auxiliary value. The image processing was performed according to the algorithm shown in Figure 5.

The performed tests of the system effectiveness showed that a standard PC is able to process the images with a frequency of 15 Hz. However, at the current stage, due to the fact that the assessment of the quality of the separation was performed as an independent task not included directly in the separation process, this value was sufficient.

## 4. Calibration of the System

The developed vision system has been calibrated on the dedicated material consisting of red and white samples. As an example of a difficult pair of polymers the following materials were selected and prepared for the tests: PMMA (red) and PS (white).

To calibrate the developed system the sample materials were placed in two separate zones containing approximately three ROIs, and then the control parameters of the image processing algorithm were manually tuned to obtain a satisfactory level of filtration for each material. Due to differences in the shape and shading of material pieces in the ROIs, the proposed approach determined the filtration parameters based on specific ranges of H, S and V colour components, defined by min and max values of each H, S, V. The determined values of the control parameters are listed in Table 1.

**Figure 6:**
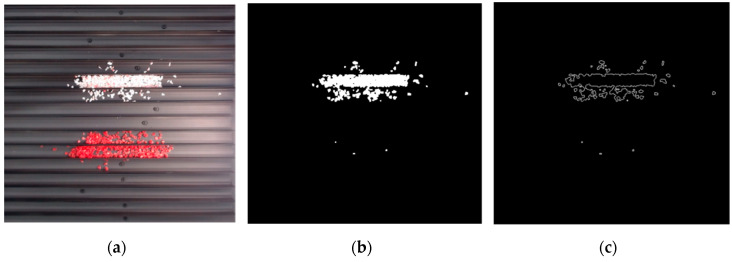
Raw image (**a**), effect of filtration (**b**) and calculated dispersion (**c**) for determined ranges of HSV parameters for PS.

**Figure 7:**
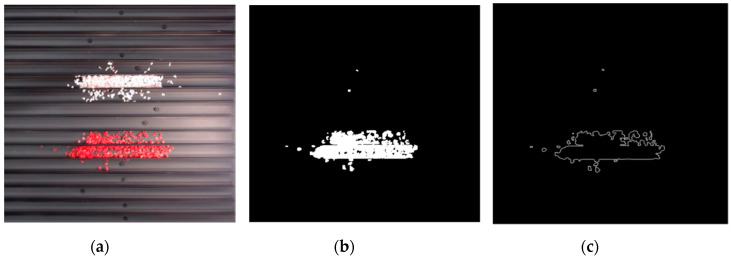
Raw image (**a**), effect of filtration (**b**) and calculated dispersion (**c**) for determined ranges of HSV parameters for PMMA.

The effect of filtration of a raw image for the studied materials has been shown in Figure 6 and Figure 7, respectively. Columns (a–c) of Figure 6 and Figure 7 present a raw image, the effect of filtration and auxiliary dispersion of the processed image, respectively.

In order to calibrate and verify the proposed method and developed vision system, the tested materials were weighed and placed in subsequent collectors in such a way that 100% of a given material would be placed in the farthest collectors. The materials were mixed in the following proportions: 100–0%, 90–10%, 80–20% … 10–90%, 0–100%. The materials were mixed by weight. Next, the developed vision system was employed to estimate the number of PMMA and PS materials in each ROI by visual assessment. Two cases were examined, i.e., with and without the morphological operation of dilatation in the considered image processing. The results are shown in Table 2.

Since the applied morphological operation of dilatation leads to sharpening the edges of detected items, it is widely employed in many applications of vision systems [5,6]. Nevertheless, in the studied case it has been observed that the employment of dilatation operation causes an additional error in the target measurements. Therefore, it was decided not to use the morphological operator.

**Figure 8:**
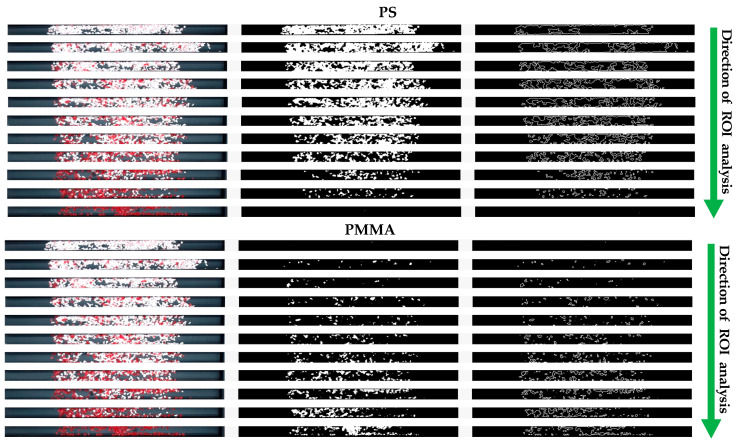
Sequence of evaluation of the sample ROIs and materials.

In the proposed algorithm, the process of assessing the amount of the specific material in each collector was carried out automatically for the subsequent segments (ROIs) and both materials, according to the order indicated in Figure 8.

**Figure 9:**
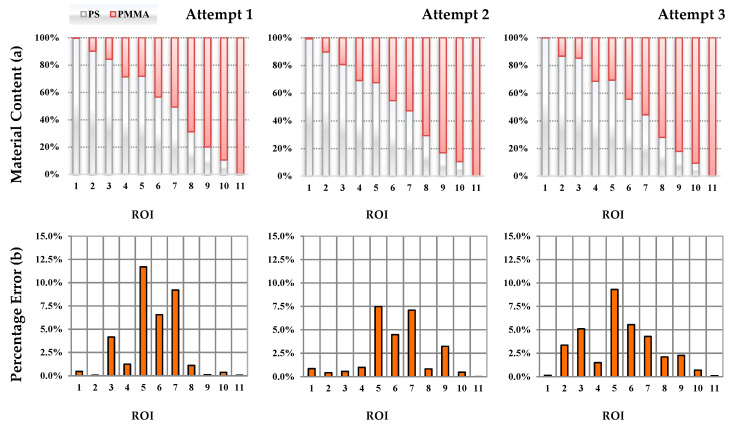
Percentage of material (**a**) and error in estimation (**b**) in chosen segments (three attempts).

Three measurement attempts were conducted. At each attempt, the material in individual collectors was randomly dispersed. The percentage contents of PS and PMMA materials in each ROI were determined and are shown in Figure 9.

The values of an error with respect to the base values were determined for each attempt. Its maximum value was for Attempt 1 and was equal to 12%. It should be remembered that the results read from the camera refer to the samples mixed by weight.

## 5. Tests of the System

**Figure 10:**
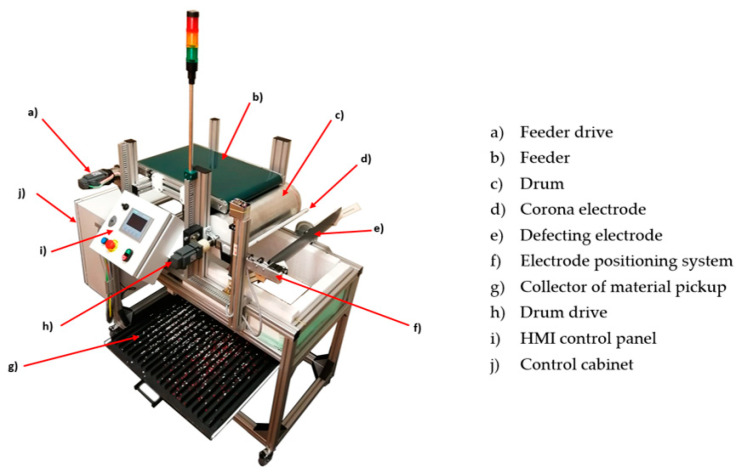
Prototype electrostatic separator.

The effectiveness of the developed vision system for studies on electrostatic separation was examined on a dedicated test stand shown in Figure 10. The test stand was designed using professional CAD methods allowing one to predict the shape of every part and any dimensions of the experimental stand (ES). The backbone frame of the construction is based on prefabricated aluminium profiles and is assembled as one structure. The application of prefabricated aluminium profiles gives the possibility to introduce any changes to connections and corrections of the main components’ positions, if needed. The proposed ES was built in a modular way. The feeder can be easily converted into a free-fall hopper, a vibration feeder device, or a tribocharging bed. The device allows for adjusting the amount of material fed. The next main part of the system is a belt conveyor which is responsible for transporting input material from the feeder to the rotating drum. The conveyor is driven by a precise industrial servo motor with a mechanical gear. The application of an advanced industrial servo drive allows for precise control of the linear speed of the belt.

The test stand is equipped with corona and deflection high voltage electrodes. The corona electrode has the ability to move along two coplanar axes to the drum base. The use of the stepper motor enables maintaining the desired position during the separation process. The deflection electrode has also the ability to change positions and rotation (manually). In the case when input material is tribocharged, the corona electrode can be removed and its automated positioning system can be utilized to control the localization of the deflection electrode without manual operations. In other words, the test bench can operate in two modes, i.e., as a typical drum type separator (corona charging mode) and as a semi-freefall type separator when the corona electrode is removed and material is tribocharged using an external device (tribocharging mode).

The effectiveness of the proposed vision system was examined on two sample tests performed on the test stand. The tests were conducted for the corona charging and tribocharging modes for the mixture of PS and PMMA material samples. The parameters of the separation process (voltage value, the position of the electrodes, and drum and feeder speeds) were experimentally tuned to obtain a separation effect. The quality of the separation, i.e., the location of materials in a material pickup was evaluated using the developed vision system. The raw and extrapolated images as well as designated contours employing a dispersion operator for the test in the corona charging mode are summarized in Table 3.

**Figure 11:**
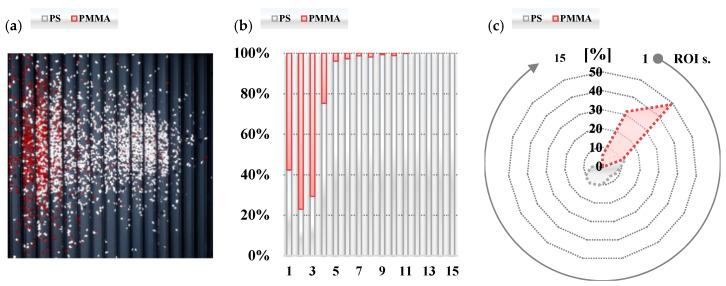
Results of processing the raw image (**a**); percentage material content in each ROI (**b**) and percentage material content related to total amount of each material (**c**) for the corona charging mode.

The developed vision system allowed us to determine numeric data about the percentage content of the material in each ROI—see the bar plot shown in Figure 11b. However, because the percentage content of the material is calculated for each ROI independently, the bar plot in Figure 11b does not provide exact information about separation quality. To mitigate this problem the amount of the material in a particular ROI was related to the total amount of the material measured in all ROIs during the test. To express the separation quality, the result of percentage material content related to the total amount of each material is illustrated in the radar type plot shown in Figure 11c.

**Figure 12:**
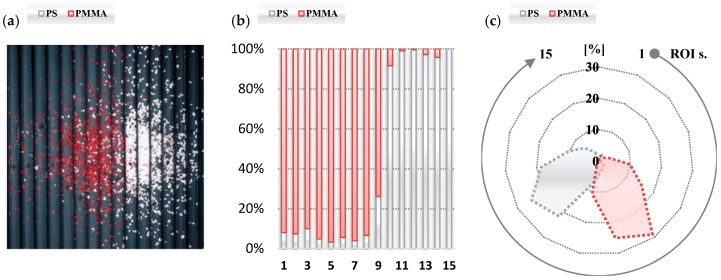
Results of processing the raw image (**a**); percentage material content in each ROI (**b**) and percentage material content related to total amount of each material (**c**) for the tribocharging mode.

The second test was performed to examine the effectiveness of the proposed vision system while the separator was in the tribocharging mode. The results are presented in an identical way as for the test of the corona charging mode and summarized in Table 4 and Figure 12, respectively.

Analysing the results, it can be observed that the proposed vision system allowed one to assess the effectiveness of the separation process. Comparing the radar plots shown in Figure 11 and Figure 12, it can be noted that the quality of the separation in the tribocharging mode during the performed tests is much higher than in the case when materials are charged by the corona discharge effect. Further analysis of the obtained data allowed us to define the factor describing separation effectiveness.

## 6. Discussion

This paper describes two approaches for the evaluation of material content in ROIs, which were tested during the experimental stage. The objective of the first approach was to determine the number of pixels in a specific colour and, then, on its basis and after conversion, to define the percentage content of specific materials in each ROI. In the second approach the evaluation of material content was based on determining the number of closed contours. On the basis of the conducted analyses, it can be stated that more accurate information about material content can be obtained based on the data derived from the number of pixels in a specific colour. In the case of agglomerated plastic particles, we could see that the particles tend to form closed contours, which distorts results and thus negatively affects the evaluation. Therefore, the final testing of separation effectiveness was based on the first method.

Based on the obtained results it can be stated that the effectiveness of the proposed vision system has been confirmed. It has been demonstrated that the developed system provides a simple and fast method to assess the quality of electrostatic separation which can be applied to research on the optimization of the process of the electrostatic separation of plastic waste. However, it should be highlighted that the proposed approach is effective when the separated materials differ in colour. It means that the proposed system can be useful only for the assessment of the separation quality of sample materials having specific colours. Nevertheless, the data about the quality of separation of the sample materials allows us to optimize the separation process and can next be applied to the target solution for separating the same materials of different colours. The key advantage of the proposed method is a significant reduction of the time needed to assess the quality of the separation during the stage of the optimization of the separation process.

According to Grigorescu et al. [30], the most commonly used techniques applied for the identification of polymers in the waste stream are spectroscopic analyses (especially Fourier transform infrared spectroscopy (FTIR)), density analysis, gel permeation chromatography (GPC), melt flow index (MFI), dilute solution viscosity (DSV), thermal analysis, including differential scanning calorimetry (DSC) and thermogravimetry (TGA). The separation of specific polymeric waste, containing brominated flame retardants, may be realized by more advanced techniques such as Raman spectroscopy [31,32]. All the mentioned methods are laboratory offline or online analyses that require more work time, the necessity of qualified personnel engagement, as well as, in most cases, preparation of samples before testing. Considering that, even if measuring devices are adapted to perform quick analyses, due to the random geometry of the obtained samples and the lack of information about their physical properties, they often require preliminary adaptation, including drying, dissolving or melt processing. All these preliminary sample preparation operations significantly extend the analysis of the current series of separated polymer materials [30].

Out of the all mentioned methods that allow for high-quality identification of polymer, the FTIR and DSC techniques deserve special attention. The main disadvantage of both methods is a complicated handling procedure as well as the possibility of signal overlapping, resulting in, e.g., formation of polymeric blends. From the point of view of fast-tracking, methods used during the industrial separation process, the measurement time and cost are more important than measurement accuracy. While the FTIR spectroscopic analysis performed with the use of an apparatus equipped with a sufficiently large database is an excellent tool for material identification, the performance of the test along with the preparation of the sample for testing and cleaning of the sample cells may take up to 20 min [30]. When using thermal analysis (DSC), the measurement takes about 1.5 to 2 h depending on the polymer type. In the case of analysing amorphous polymers, except for the glass transition inflexion of the DSC curve, no signal that represents the exo- and endothermic changes corresponding to the process of crystallization and melting will be obtained, which could be the basis for identification [33]. Moreover, the measurement ambiguity may also result from the widespread use of additives and modifiers, changing the thermal properties of semicrystalline polymers and their composites, as well as inappropriate preparation of the sample [34].

The assessment of separation effectiveness by the proposed vision system is about dozen times faster than the discussed above analytical methods. This enables us to perform optimization of the separation process by testing many variants of the parameters affecting its quality, such as electrodes positions, feeder and drum speeds as well as HV value and parameters of the tribocharging process. However, it should be emphasised that the evaluation of separation quality by a vision system is not as precise as by the FTIR or DSC methods [30,33]. The estimated error (see Figure 8) is up to 10% depending on contents of the material in the ROIs. Nevertheless, for the needs of optimization of the separation process parameters such precision is sufficient assuming that the research is conducted in a comparative manner and the main focus is paid to ROIs in which the amount of one material dominates over the other. The estimated error for such cases is less than about 5%.

## 7. Conclusions

The paper presents an effective method employing a dedicated vision system for the assessment of the quality of the electrostatic separation of plastics mixtures. The elaborated system was calibrated and tested on a mixture of poly(methyl methacrylate) (PMMA) and polystyrene (PS). It has been demonstrated that vision systems can be successfully employed in the research on electrostatic separation of plastics, providing a fast and accurate method for the assessment of separation process purity and its effectiveness. It should be noted that the proposed method not only can be applied for assessing the electrostatic separation results but also is useful in the research employing other methods of plastics separation.

Summarizing the results of the tests of the electrostatic separation process described in the article, we can point out that the quality of the separation in the tribocharging mode was much higher than in the case when materials were charged by the corona discharge effect. Nevertheless, it should be emphasised that the discussed results relate to the initial tests of the vision system and don’t concern the research on the optimization of the electrostatic separation process.

To improve the accuracy of the proposed method for the separation quality evaluation by a vision system, different lighting systems can be applied. Preliminary testing of using ultraviolet light to increase the ability to discriminate between materials is under way and has given promising results so far.

The method can be extended to separate materials with a higher number of colours. In general, it would allow researchers to simplify the preparation process of sample materials. However, the tuning process of parameters of the discrimination algorithm will be more complex and time consuming. Moreover, an increase in the number of analysed colours of materials can also lead to an increase of assessment error, especially when colours representing different materials are close to each other in the HSV colour space.

## Figures and Tables

**Table 1:** Filtration parameters defined by HSV ranges.

Parameter\Material	PMMA (Red)	PS (White)
*H_min_*	0	167
*H_max_*	179	216
*V_min_*	138	123
*V_max_*	255	255
*S_min_*	0	122
*S_max_*	28	255

**Table 2:** Effect of morphological operation on processed images.

	Raw Image of the Material	Extrapolated Image of the Material	Designated Contours (Dispersion)
**PS without Dilatation**	** 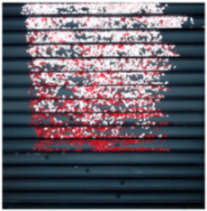 **	** 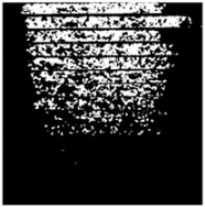 **	** 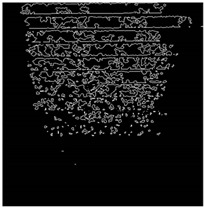 **
**PS with dilatation**	** 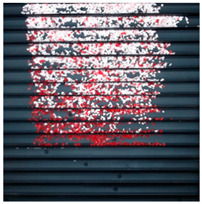 **	** 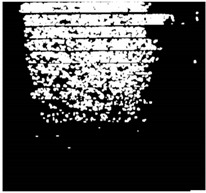 **	** 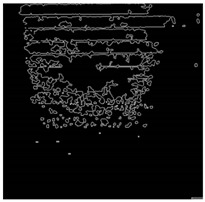 **
**PMMA without Dilatation**	** 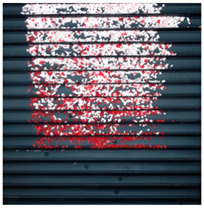 **	** 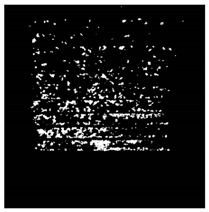 **	** 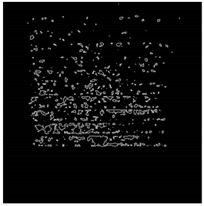 **
**PMMA with Dilatation**	** 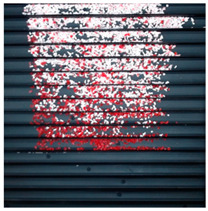 **	** 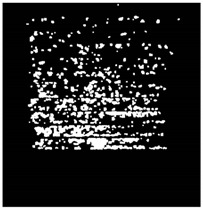 **	** 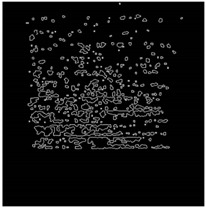 **

**Table 3:** Tests with separator—corona charging mode.

	Raw Image	Extrapolated Image	Designated Contours
**PS**	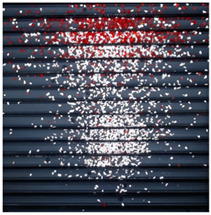	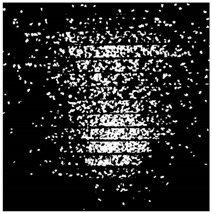	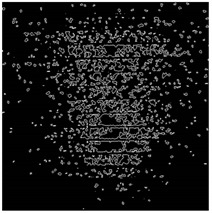
**PMMA**	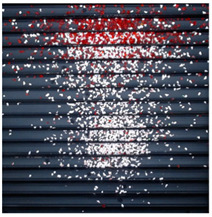	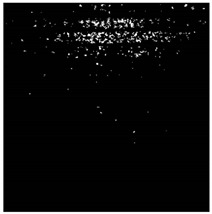	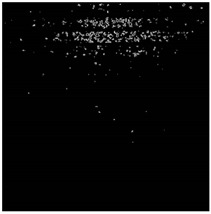

**Table 4:** Tests with separator—tribocharging mode.

	Pure Image	Extrapolated Image	Designated Contours
**PS**	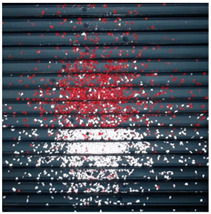	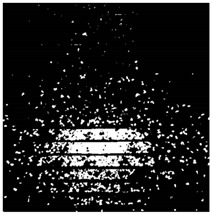	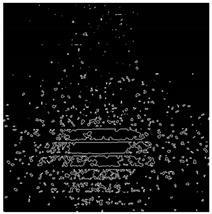
**PMMA**